# Intestinal Diseases

**Published:** 1895-01-12

**Authors:** 


### Progress in Medicine.

### INTESTINAL DISEASES.

**Summer Diarrhœa.**—Dr. Spottiswoode Cameron, at the annual meeting of the British Medical Association, referred to the increased mortality in the large Yorkshire towns from summer diarrhœa. The maxima of deaths occurred in the second week of July and the fourth week of August. Dr. Cameron considers that many of the cases of death in Leeds that he investigated were dependent upon "preventible circumstances" in connection with the sewage of the houses. The middens and ashpits were emptied and gullies cleansed more frequently in one district, and that district was the only one in which the death-rate from diarrhœa was lower in 1893 than in 1892.[1] Dr. Cameron gives other details in a paper read before the Yorkshire branch of the British Medical Association.[2] Of 605 fatal cases of diarrhœa 60 per cent. occurred in houses either without drains, or with drains not properly severed from the sewer, and one-fifth of the remainder (making 73 per cent.) had other sanitary defects. As to the question of milk forming the vehicle by which infection takes place, there were 153 cases of death under one year, but of those only 18 per cent. were entirely breast fed.

**Guava in Diarrhœa.**—The leaves of the white guava of the West Indies and Java are praised by Dr. Hugel in the treatment of diarrhœa.[3] It is stated that the aborigines of Java assert that the remedy is of value in Asiatic cholera. Dr. Hugel found a guava infusion more useful than any other method of treatment in cholera infantum and cholera nostras. Seventy-five grains of the guava leaves are added to two ounces of boiling water, and a teaspoonful to a tablespoonful (according to the age of the patient) given every hour or every other hour. It was also found useful in the treatment of the diarrhœa of typhoid fever and of phthisis.[4]

**The Treatment of Dysentery.**—Another drug is praised by Schwarz, of Constantinople, in the treatment of dysentery. Dr. Schwarz for a long time was puzzled by the remarkable results obtained by quack doctors, and at length succeeded in discovering the constituents of their compound. The principal ingredient is myrobalana, the fruit of an indigenous tree, Termenalia chebula, made up with pelleterine, extract of rosemary, and extract of pomegranate rind.[5]

**The Etiology and Propagation of Dysentery.**—At the Hygiene Congress at Buda Pesth Prof. Celli, of Rome, read a paper upon the amœba of dysentery. He considers it allied to the plasmodium malariæ, and calls it a sporozoon, not an amœba. He, however, thinks that it does not play an important part in the production of dysentery, since he discovered it only twelve times in thirty-four cases examined[6]. In a leading article in the *British Medical Journal*, March 17th, 1894, the propagation of dysentery by drinking water is dwelt upon. It is mentioned that as long ago as the days of Hippocrates water was supposed to be the source of infection, a supposition now acknowledged to be supported by numerous facts. Dr. George Turner[7] issues an interesting report bearing upon this subject. Several outbreaks of diarrhœa and dysentery have occurred from time to time at the Suffolk County Lunatic Asylum, and one occurring in the autumn of 1893 was investigated. In that outbreak there were forty-two cases of diarrhœa and thirty-two of dysentery. By a process of exclusion, Dr. Turner decided that the water supply was probably the source of infection. The water was derived from two wells sunk in chalk lying at depths of 305 and 350 feet. Carefully constructed though they were, the water appeared to have suffered contamination from surface soakage, one well being affected more than the other. The water of this well contained 11·3 grs. of chlorine, and microscopic examination of the sediment showed vegetable *débris*, bacteria, and spirilla, objects foreign to chalk water. Certain organisms were also found that bore a resemblance to the amœba of dysentery.

Very interesting facts are also mentioned by Dr. Gresswell, of Melbourne.[8] An outbreak of dysentery occurred in that city in November, 1892. In a block of 185 houses 140 received one water supply, and all the dysentery occurred amongst those 140. In 53 houses the water had been habitually boiled before use, and only 1·8 per cent. were invaded. At 68 houses the water was unboiled, and here 50 per cent. suffered. In five houses it was filtered, and in these there was only one person attacked. Further investigations showed that there were numerous sources of water contamination.

**Anchylostomiasis.**—Dr. Sandwith,[9] of Cairo, publishes an exhaustive paper upon the anchylostomum duodenale. He mentions that since 1878 the disease produced by this intestinal worm has been found spread over the North of Italy, in Sardinia and Sicily, amongst the miners in Spain and Hungary, among the brickmakers in Germany, Belgium, and the Netherlands. It has also been noticed in various districts in France. Above the latitude 47° N. it can only flourish in the sheltering warmth of mines. In warmer climates it is met with, and the disease has been noticed in various districts in Asia and Africa, as well as in the Southern States of North America, and in Bolivia and Peru. In Egypt it is widespread, and it is interesting that the symptoms produced by the marked anæmia of this disease were described by the Egyptian physicians of 3450 B.C. Dr. Sandwith has had 402 cases under his care; of these only three were in the female sex. The commonest period of life for the disease is between thirty and forty years of age, but there were 48 cases between ten and twenty and a few between six and twelve. The occupation of the sufferers is important. Of 200, in which careful notes were taken, 190 were more or less accustomed to work with their hands in damp earth. They were mainly agricultural labourers, builders of mud walls, and street scavengers. The explanation of this association of the disease with occupations of this character is that the embryos of the worm will live in damp earth for about six weeks, and for a much longer period if supplied with fresh fæces. Defæcation by the natives is performed almost anywhere, and thus the soil becomes infected. Individuals contract the disease by eating with earth-covered hands. Dr. Sandwith goes fully into the various features of the disease. In connection with diagnosis, it is mentioned that there is seldom any difficulty in recognising it on account of the marked anæmia, but confirmatory evidence can be obtained by careful examination of the fæces. After repeated washings with water the parasite can be discovered under the microscope. The duration of the disease is sometimes long; for example, one patient said that he had suffered for fifteen years. Of cases under the care of Dr. Sandwith 8 per cent. died. Death was attributed to the consequences of the anæmia in the majority of instances, but the fatal result may also have been partly due to inanition following impaired digestion, consequent upon pathological changes in the duodenum and jejunum set up by the presence of the worms. The treatment of the disease appears to be simple, although, unfortunately, Dr. Sandwith thinks that in two instances it was responsible for the death of the patient. It consists in giving four grammes of thymol, at two hours' interval, the morning after an aperient has been given. Most of the worms come away after the first dose, but the drug may need to be repeated until two, three, four, or five doses have been given. In 184 cases Dr. Sandwith was able microscopically to prove the absence of the parasite in the fæces after treatment. The sulphate of iron was found the most useful drug for the treatment of the anæmia. Dr. Sandwith thinks that the disease might be stamped out by compelling the villagers to dig trench latrines, and to use those latrines alone. It would be difficult, however, to enforce regulations upon such a point, as it would also be to impress them with the necessity of washing their hands before eating.

**Treatment of Tapeworm.**—Dr. Leslie Ogilvie considers that one drachm of the extract of male-fern is not sufficient to cause the death of a tapeworm. He thinks a second dose should be given at an hour's interval. He also considers that castor oil is not the best aperient to give the night before, since it does not clear away mucus from the intestine. Sulphate of magnesia and tincture of jalap he thinks answered better. Castor oil, however, may be given to bring away the worm two hours after the administration of the second dose of the extract of male-fern. Hunting for the head is rendered less disagreeable by using liquor potossæ permanganatis freely as a deodorant.

[1] Brit. Med. Journ., August 11th, 1894. [2] Lancet, June 30th, 1894. [3] Med. Week, August 10th, 1894. [4] Med. Week, August 10th, 1894, [5] Prov. Med. Journ., Feb., 1894, and Internatl. Klin Rundschau, 1893. [6] Brit. Med. Journ., Sept. 22nd, 1894. [7] Pract., Sept., 1894. [8] Pract., Sept., 1894. [9] Lancet, June 2nd, 1894.

